# Coacervate‐Enhanced Deposition of Sprayed Pesticide on Hydrophobic/Superhydrophobic Abaxial Leaf Surfaces

**DOI:** 10.1002/advs.202300270

**Published:** 2023-04-20

**Authors:** Liangchen Zhang, Jie Wang, Yaxun Fan, Yilin Wang

**Affiliations:** ^1^ CAS Key Laboratory of Colloid Interface and Chemical Thermodynamics CAS Research/Education Center for Excellence in Molecular Sciences Beijing National Laboratory for Molecular Sciences Institute of Chemistry Chinese Academy of Sciences Beijing 100190 P. R. China; ^2^ University of Chinese Academy of Sciences Beijing 100190 P. R. China

**Keywords:** abaxial leaf surface, coacervates, deposition, superhydrophobic, sustainable agriculture

## Abstract

Deposition of high‐speed droplets on inverted surfaces is important to many fundamental scientific principles and technological applications. For example, in pesticide spraying to target pests and diseases emerging on abaxial side of leaves, the downward rebound and gravity of the droplets make the deposition exceedingly difficult on hydrophobic/superhydrophobic leaf underside, causing serious pesticide waste and environmental pollution. Here, a series of bile salt/cationic surfactant coacervates are developed to attain efficient deposition on the inverted surfaces of diverse hydrophobic/superhydrophobic characteristics. The coacervates have abundant nanoscale hydrophilic/hydrophobic domains and intrinsic network‐like microstructures, which endow them with efficient encapsulation of various solutes and strong adhesion to surface micro/nanostructures. Thus, the coacervates with low viscosity achieve high‐efficient deposition on superhydrophobic abaxial‐side of tomato leaves and inverted artificial surfaces with a water contact angle from 170° to 124°, much better than that of commercial agricultural adjuvants. Intriguingly, the compactness of network‐like structures dominantly controls adhesion force and deposition efficiency, and the most crowded one leads to the most efficient deposition. The tunable coacervates can help comprehensively understand the complex dynamic deposition, and provide innovative carriers for depositing sprayed pesticides on abaxial and adaxial sides of leaves, thereby potentially reducing pesticide use and promoting sustainable agriculture.

## Graphical abstract

A general approach has been first proposed to subtly adjust crowding inside coacervates by changing surfactant chain length, and the crowding, i.e., the compactness of network‐like structures, can controllably improve dynamic deposition of water‐based droplets on inverted superhydrophobic surfaces and abaxial side of leaves after bottom‐up spraying without the assistance of high viscosity, low surface tension and rapid molecular diffusion.

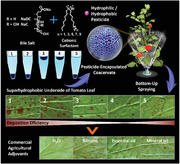

## 1 Introduction

Pesticide spraying is a very essential but hard task in agriculture to protect plants from harmful organisms. In nature, more than half of crop leaves display diverse hydrophobic characteristics on both their adaxial and abaxial sides.^[^ ^1^ ^]^ Some of them are intrinsically superhydrophobic, like major food crops (wheat and rice), or even become highly hydrophobic after being infected with pathogen,^[^ ^2^ ^]^ which causes up to 60% of agrochemicals to be lost due to undesired bouncing and splashing behaviors on crop leaves. Particularly, many pests and diseases, such as Whiteflies, Adult Tetranychus urticae, and Red spider, emerge on the abaxial side of their leaves, leading to serious foliar damage and reduced yields of many fruits and vegetables.^[^ ^3^ ^]^ Bottom‐up pesticide spraying is the most direct and efficient method for targeting the pests and diseases on the abaxial side of leaves, but the downward rebound and gravity of the droplets make the deposition exceedingly difficult, in particular on the hydrophobic/superhydrophobic leaf underside. This dramatically exacerbates agrochemical waste, resulting in the pollution of soil or water and threatening ecological security and human safety. Therefore, pesticide carriers are imperatively desired to overcome rebound and gravity so as to achieve highly efficient deposition and retention on both adaxial and abaxial sides of leaves after high‐speed spraying.

In recent years, various versatile strategies have been established to improve the deposition and retention of impacting droplets on hydrophobic/superhydrophobic surfaces.^[^ ^3^, ^4^ ^]^ However, they were all targeted on the upper surfaces, while the situation for the underside of the surfaces has not been involved. Even on the upperside, rebound and splash of sprayed pesticide droplets are still very difficult to be inhibited because of the very short contact time (several milliseconds) and low‐surface‐tension enhanced retraction velocity after high‐speed impact,^[^ ^5^ ^]^ as well as the water‐repellent feature of leaf surfaces.^[^ ^1^ ^]^ In impact, the mainstream approaches to dramatically inhibit splash on such surfaces are delaying drop retraction through polymer additives^[^ ^4c,d^ ^]^ or strengthening interaction with surface micro/nanostructures by appropriate self‐assembled structures of surfactants.^[^ ^4e–i^ ^]^ Particularly, these surfactant aggregates well‐used in suppressing droplet rebound can also exhibit good performance on the sequestration and penetration of pesticides, which were pioneeringly proposed by our groups and are most likely to solve the unavoidable organic solvent usage in current pesticide carriers. Inspired by these, subtly modulating microstructures of surfactant aggregates are expected to obtain robust pesticide carriers aiming at pests and diseases on the abaxial side of plant leaves, which will possess the comprehensive advantages of water solvent, good liquidity, easy to spray, well encapsulation of diverse solutes, and strong adhesion on hydrophobic/superhydrophobic substrates.

Coacervates, liquid‐like microdroplets, provide a promising means to spontaneously sequester a wide range of chemicals, endowing them with wide application in reaction acceleration, wastewater treatment, drug delivery, cosmetic formulations, etc.^[^ ^6^ ^]^ Recently, coacervates have also been encapsulated inside liposomes through several approaches,^[^ ^7^ ^]^ making them also useful as crowed artificial cells or drug delivery. Especially for surfactant‐based coacervates, their disordered bicontinuous or network‐like internal microstructure facilitates the functional loading capacity for hydrophilic/hydrophobic substances simultaneously.^[^ ^8^ ^]^ Meanwhile, coacervates have exhibited superior adhesion ability to wet solid surfaces in many marine organisms.^[^ ^9^ ^]^ Their nanosized network‐like internal microstructure also results in high affinity to the superhydrophobic surface and superior deposition performance after high‐speed impact,^[^ ^4h,i^ ^]^ attributed to the interaction with the surface micro/nanostructures to pin the contact line during the impact process. Therefore, we expect that surfactant coacervates could efficiently deposit bottom‐up sprayed droplets on the inverted surfaces, inhibiting the effects of gravity and rebound.

In this work, we develop a class of surfactant coacervate systems to improve the droplet deposition on inverted surfaces with different hydrophobicity. The coacervates are generated by the spontaneous phase separation of bile salts with a series of dialkyldimethylammonium bromide surfactants. Due to the tunable nanosized network‐like structure, the coacervates can efficiently hold hydrophilic/hydrophobic dyes, biomacromolecules, and pesticides, and exhibit superior affinity to the micro/nanostructures of superhydrophobic surfaces. The superior affinity endows the sprayed droplets with high‐efficient deposition on the inverted surfaces of various hydrophobic characteristics (water contact angle from 170° to 124°) and the superhydrophobic abaxial side of tomato leaves against rebound and gravity. Our study provides a feasible strategy to prevent diseases and pests on the adaxial and abaxial sides of leaves by the controllable environmental‐friendly pesticide carriers.

## 2 Results and Discussion

**Figure 1:**
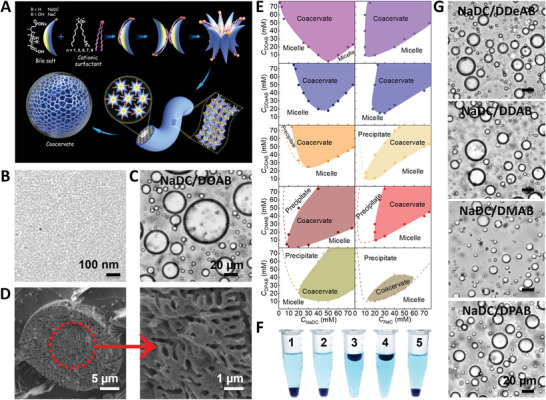
Coacervation in the mixtures of bile salts and dialkyldimethylammonium bromide. A) Schematic illustration of phase transitions of NaDC/DXAB and NaC/DXAB mixtures. B) Cryo‐TEM image of rodlike micelles in 20 mm NaDC/20 mm DOAB mixed solution. C) Bright‐field microscopy and D) cryo‐SEM image of coacervate microdroplets in 30 mm NaDC/30 mm DOAB mixed solution, wherein the rightmost is a magnified image of the nanosized network‐like microstructure. E) Phase boundaries of NaDC/DXAB and NaC/DXAB mixtures. DXAB = DOAB, DDeAB, DDAB, DMAB, and DPAB. F) Optical images of coacervates for NaDC/DOAB (1), NaDC/DDeAB (2), NaDC/DDAB (3), NaDC/DMAB (4), and NaDC/DPAB (5) after phase separation in 1.5 mL centrifuge tubes. The bottom phase is termed as the condensed phase for NaDC/DOAB, NaDC/DDeAB, NaDC/DPAB, and referred to the dilute phase for NaDC/DDAB and NaDC/DMAB. G) Bright‐field microscopy image of coacervate microdroplets in 30 mm NaDC/30 mm DXAB mixed solutions. Scale bar = 20 µm and DXAB = DDeAB, DDAB, DMAB, and DPAB.

Charge neutralization and multiple binding sites play a key role in forming surfactant coacervates.^[^ ^7^ ^]^ Sodium deoxycholate (NaDC) and sodium cholate (NaC) as unique facial amphiphiles consist of a saturated tetracyclic steroidal skeleton with two or three hydroxyl groups (–OH) and a flexible isopentanoic side chain with a carboxylic end group.^[^ ^9^ ^]^ Dialkyldimethylammonium bromide (DXAB, alkyl = octyl (DOAB), decyl (DDeAB), dodecyl (DDAB), tetradecyl (DMAB), and hexadecyl (DPAB)) contains one charged headgroup and two hydrophobic chains, showing strong self‐assembling ability and adjustable capacity. Combining their structural advantages, we construct coacervates in the mixture of NaDC or NaC with oppositely charged surfactants (DXAB = DOAB, DDeAB, DDAB, DMAB, and DPAB) by subtly adjusting their binding affinity (**Figure** **1**A).

NaDC and NaC are water soluble at room temperature. When the concentration is beyond the critical micelle concentration (CMC, 4.0 and 11.0 mm) of NaDC and NaC in an aqueous solution,^[^ ^10^ ^]^ they form micelles in a back‐to‐back packing way with the hydrophobic faces oriented toward the micellar inside. Meanwhile, the DXAB surfactants are prone to form vesicles.^[^ ^4g^ ^]^ By blending the two components and increasing the total concentration at the equal molar ratio, the rodlike micelles are formed, with a few tens of nanometers long and more than ten nanometers wide (Figure 1B). Then the rodlike micelles transfer into coacervate showing turbid dispersions of 10–50 µm‐sized spherical microdroplets with nanosized network structure (Figure 1C,D). Moreover, the coacervate microdroplets are generally formed and stable over a wide range of the NaDC/NaC and DXAB concentrations as depicted in phase boundaries (Figure 1E), which are derived from the preparation and characterization of nearly one hundred samples for each mixed system.

Strong electrostatic binding, hydrophobic interactions, and hydrogen bonding in the mixed NaDC/NaC and DXAB systems drive the coacervation. Upon mixing anionic NaDC/NaC with cationic DXAB, the electrostatic binding between the oppositely charged headgroups of the two surfactants and the strong hydrophobic interaction among the hydrophobic parts of NaDC/NaC and the double alkyl chains of DXAB promote the micellization of NaDC/NaC and the micellar growth. With increasing the concentration, the hydrophobic interaction is getting stronger, and the hydrophobic domain becomes much bulkier compared with that of hydrophilic headgroups, causing the aggregate spontaneous curvature to become small and even negative. The negative Gaussian curvature is beneficial to the transition from rodlike micelles to coacervates.^[^ ^11^ ^]^ In addition, the large number of hydroxyl groups of NaDC/NaC exposed to the micellar surface produces strong hydrogen‐bonding interaction, which not only evokes rodlike micelles to align side by side and grow wider, but also facilitates the association of the rodlike micelles with each other. Thus, the coacervates are formed with low zeta potential (Table S1, Supporting Information) and with crowding inside, that is, internal nanosized network‐like microstructure. The longer alkyl chain length leads to stronger intermolecular hydrophobic interaction and more steric resistance, which enhances coacervation coupled with the stronger release of counterion and dehydration, resulting in larger precipitation regions in the concentration ranges studied.^[^ ^12^ ^]^ Besides, NaDC has one less hydroxyl group than NaC, thereby the hydrogen bonds between different rodlike micelles are slightly weakened, and the appropriate binding affinity is more beneficial for coacervation.

Intriguingly, the continuous changes in the alkyl chain length of the surfactant DXAB cause a fluctuation in the density of the condensed coacervate phase, that is, from the bottom phase to the upper phase and then to the bottom phase again (Figure 1F). But all the coacervates show turbid dispersions of similar 10–50 µm‐sized spherical microdroplets (Figure 1C,G and Figure S1, Supporting Information). It is inferred that the crowding degree inside the coacervates is adjustable, which could be used to regulate the encapsulation of hydrophobic/hydrophilic guest molecules and the deposition of sprayed droplets on the inverted surfaces of various hydrophobicity degrees.

**Figure 2:**
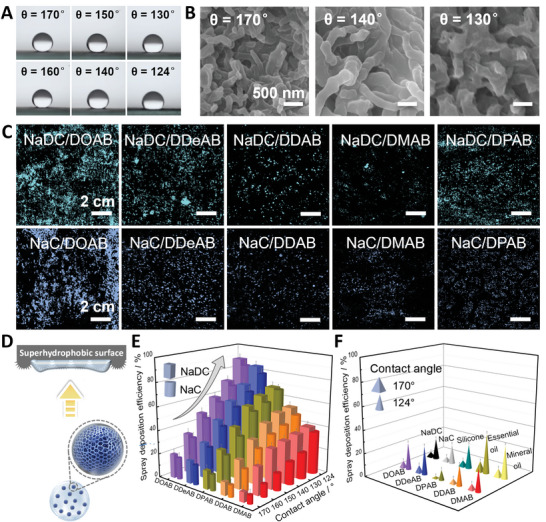
Coacervate droplets deposit on hydrophobic/superhydrophobic undersides after bottom‐up spraying. A) Water contact angle (*θ*) on modified filter paper with different hydrophobicity. B) SEM images of the morphology of representative surfaces (*θ* = 170°, 140°, and 130°). Scale bar, 500 nm. C) Optical images of dye‐encapsulated coacervate droplets depositing on superhydrophobic filter paper (*θ* = 150°) after bottom‐up spraying. The coacervates are formed by 30 mm NaDC/30 mm DXAB and 30 mm NaC/30 mm DXAB. Scale bar, 2 cm. D) Schematic diagram of the bottom‐up spray. The distance between the sprayer and the surface is fixed at 20 cm. The velocity of the spray droplets reaching the hydrophobic/superhydrophobic surface is about 1.80 ± 0.10 m s^−1^. E,F) The deposition efficiency of (E) coacervate droplets of 30 mm NaDC/30 mm DXAB and 30 mm NaC/30 mm DXAB, and (F) controls (60 mm NaDC, 60 mm NaC, 60 mm DXAB and three types of commercial agricultural adjuvants, including 1.3 mg L^−1^ silicone, 1.0 mg L^−1^ essential oil, and 1.0 mg L^−1^ mineral oil) on hydrophobic/superhydrophobic surfaces after bottom‐up spraying. In all images, DXAB = DOAB, DDeAB, DDAB, DMAB, and DPAB.

Hydrophobicity or superhydrophobicity is one of the most striking features of many plant leaves, both for their adaxial and abaxial sides.^[^ ^1^ ^]^ By changing the types of long‐chain organosilane (octadecyltrichlorosilane and methyltrichlorosilane) and the stoichiometric ratio of organosilane to water,^[^ ^13^ ^]^ we prepared six kinds of filter papers with different hydrophobicity, which can realistically mimic the hydrophobicity of the diverse foliage and study the deposition behavior of the droplets. The contact angle of water ranges from 170° to 124° with an interval of ≈10° (**Figure** **2**A), which is produced by the fibrous micro/nanostructures with different packing densities (Figure 2B). The more compact packing of organosilane fibers results in stronger hydrophobicity. The same method also makes similar hydrophobic modifications on glass slides and nonwoven fabrics (Figure S2, Supporting Information), and thus provides more material options for testing the deposition efficiency of water‐based pesticides on various foliage.

In order to simulate the impact behavior of water‐based pesticide droplets on the abaxial side of plant leaves, we first tested if water droplets containing the NaDC/DXAB or NaC/DXAB coacervates could enhance the deposition efficiency on the inverted modified surfaces after bottom–up spraying. Fascinatingly, all the NaDC/DXAB and NaC/DXAB coacervate dispersions display continuous and semicontinuous island‐like liquid films on the superhydrophobic filter paper (*θ* = 150°), and the liquid spots distribute around the impact point eventually (Figure 2C and Figure S3, Supporting Information) after bottom–up spraying (Figure 2D). For a given bile salt, the deposition efficiency decreases with elongating the chain length of DXAB, in the order of DOAB > DDeAB > DPAB > DDAB > DMAB, as evaluated by the weight gains of the filter paper after spraying (Figure 2E, Tables S2 and S3, Supporting Information). In particular, the coacervates with the shortest surfactant DOAB achieve optimal wettability and deposition on the superhydrophobic surface, and most of the droplets adhere to the surface with only a few small droplets bounced or splashed. Anomalously, DPAB with the longest chain length shows better deposition efficiency than DDAB and DMAB with a medium chain length. Intriguingly, this varying tendency with the change in the surfactant chain length basically accords with the density fluctuation of the condensed coacervate phase (Figure 1F).

As the inverted surfaces change from superhydrophobic to hydrophobic (*θ*, 170°–124°), the deposition efficiency for each coacervate dispersion is improved up to fivefold upon bottom–up spraying (Figure 2E). Even on the most superhydrophobic surface (*θ* = 170°), the deposition efficiency of the NaDC/DXAB and NaC/DXAB coacervates can still reach 20.2%, but the droplets containing individual NaDC, NaC, and DXAB as well as the typical commercial agricultural adjuvants (silicone, essential oil, and mineral oil) exhibit violent bouncing and splashing, resulting in extremely low deposition efficiency, less than 9.0% (Figure 2F and Table S4, Supporting Information). When the hydrophobicity is weakened to *θ* = 124°, near or above the hydrophobicity of most of the leaf undersides, the maximum deposition efficiency can reach 88.9% by the NaDC/DOAB coacervate and the minimum one still maintains 36.4% for the NaC/DMAB coacervate, which is much higher than that of a single component or commercial agricultural adjuvants. Besides, the NaDC‐containing systems generally show a better deposition than NaC‐containing systems, possibly because NaDC with one less hydroxyl group exhibits weaker hydrogen bonds and dehydration in the coacervate, so it is more beneficial to bind with various specific polar microregions at the hydrophobic/superhydrophobic surfaces and helpful for the surface wettability transition.^[^ ^4h,i^ ^]^ In brief, the above results demonstrate that the NaDC/DXAB and NaC/DXAB coacervates offer broad options, which significantly enhance pesticide deposition on inverted surfaces with different hydrophobic characteristics.

**Figure 3:**
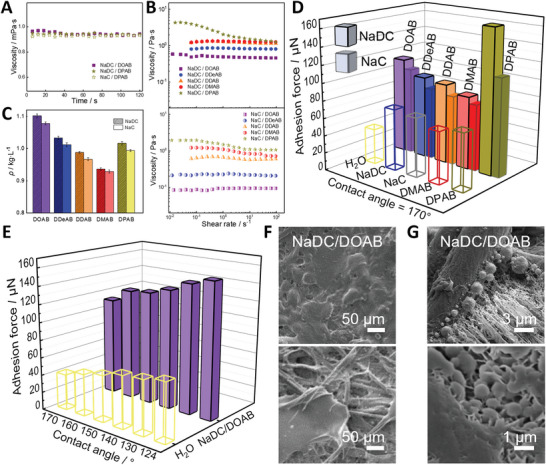
Deposition mechanism analysis of the sprayed coacervate droplets on hydrophobic/superhydrophobic surfaces against gravity. A) Steadily‐shear rheological data of the 30 mm NaDC/30 mm DOAB, 30 mm NaDC/30 mm DPAB, and 30 mm NaC/30 mm DOAB coacervate dispersions. B) Steadily‐shear rheological data and C) the density of the condensed phase of 30 mm NaDC/30 mm DXAB and 30 mm NaC/30 mm DXAB coacervates. D) Adhesion force of coacervate dispersions (30 mm NaDC/30 mm DXAB and 30 mm NaC/30 mm DXAB), micelles (60 mm NaDC or NaC), vesicles (60 mm DXAB) and pure water on the superhydrophobic filter paper (*θ* = 170°). E) Adhesion force of the 30 mm NaDC/30 mm DOAB coacervates and pure water on modified filter papers with different hydrophobicity (124° < *θ* < 170°). F) SEM and G) cryo‐SEM images of coacervate droplets (30 mm NaDC/30 mm DOAB) after bottom‐up impacting on the superhydrophobic filter paper. In all images, DXAB = DOAB, DDeAB, DDAB, DMAB, and DPAB.

How can the NaDC/DXAB and NaC/DXAB coacervates overcome the downward rebound and gravity, thereby making the prayed water droplets deposit on the hydrophobic/superhydrophobic undersides? Viscosity, dynamic surface tension, and molecular diffusion coefficient have been approved to significantly affect the dynamic impact process of droplets on the surfaces.^[^ ^14^ ^]^ Although large molecules and aggregates limit the molecular diffusion from the air/water interface and bulk solution to impact‐created solid/liquid interface, our previous work^[^ ^4h,i^ ^]^ found that they are able to inhibit the rebound of droplets on the surfaces. Herein, the coacervate is formed by bulky bile salts and DXAB with two long alkyl chains. The crowding in coacervates makes the molecules impossible to quickly move in the solution and at interfaces. The dynamic surface tension (DST) curves of the coacervate systems show a very special variation tendency with surface aging time because of the phase separation of coacervate, and the DST values are not powerful enough in inhibiting the droplet rebound on the surfaces compared with previous results^[^ ^4f,g^ ^]^ (Figure S4, Supporting Information). As for the rheological properties, their apparent viscosities of the coacervate dispersions as represented by NaDC/DOAB, NaDC/DPAB, and NaC/DPAB are just like that of water, around 1.0 mPa s (**Figure** **3**A), and the viscosity of condensed phase only slightly increases in ≈0.5–3.5 Pa s for NaDC/DXAB and ≈0.1–2.0 Pa s for NaC/DXAB with increasing longer chain length of DXAB (Figure 3B), suggesting that these coacervate systems are very easy to be sprayed. The viscosity is also not a determinative role in the high‐efficient deposition of the coacervates, either. So, there must be some other reasons for the condensed coacervate phase to control the droplet deposition on the surfaces, impelling us to turn to quantitatively determine the density and adhesion force of the condensed coacervate phase.

As mentioned above, the density of the coacervate phase decreases first and then increases again with elongating the alkyl chain length of the DXAB surfactants (Figure 3C), given that the dilute phase for all the NaDC/DXAB and NaC/DXAB mixtures shows a similar density (0.990 ± 0.005 kg L^−1^). This noncontinuous variation is different from the changing tendency of the viscosity. The coacervate viscosity is continuously enhanced with increasing the alkyl chain length of DXAB (Figure 3B) because the hydrophobic interaction among the alkyl chains is strengthened by the longer length and in turn, enforces the stronger intersection of the network‐like microstructures. However, the coacervate density is not always increased by enhancing the hydrophobic interaction of the alkyl chains. When the longer alkyl chains of the DXAB surfactants insert into the hydrophobic domains of NaDC or NaC micelles, the enhanced hydrophobic interaction may lead to compact molecular packing, but the flexibility of the co‐assembled rodlike micelles may be weakened, and thus the number of nodes per unit length may be weakened. As a result, a looser network‐like structure with a lower density is formed from DOAB to DMAB. For DPAB with the longest chains, the alkyl chain length is too long to bend over, inducing the formation of a denser network‐like structure than DDAB and DMAB. Coincidentally, the density varying tendency (Figure 3C) is consistent with the varying tendency of the deposition efficiency (Figure 2E), thereby the crowding in coacervates should take an important role in the deposition efficiency.

Meanwhile, the adhesion force of all the coacervate droplets on the most superhydrophobic surface (*θ* = 170°) is derived by measuring the advancing and receding contact angles. For a given bile salt, the adhesion force decreases as DPAB > DOAB > DDeAB > DDAB > DMAB (Figure 3D and Movie S1, Supporting Information), and the NaDC series (165.5, 111.1, 94.9, 92.1, and 83.8 µN) are correspondingly stronger than the NaC series (112.5, 100.8, 83.2, 80.1, and 76.5 µN). The varying order is consistent with that of the density except for DPAB, possibly because the condensed phase of DPAB‐containing coacervates displays high viscosity and surface tension concurrently. As controls, the adhesion force of individual components and pure water is much weaker than the corresponding coacervate dispersion, and even too low to be measured for DOAB, DDeAB, and DDAB. That is to say, the coacervates with a more crowded network‐like microstructure display larger adhesion force and result in higher deposition efficiency on the superhydrophobic surface. In addition, the adhesion force is improved by decreasing the surface hydrophobicity (Figure 3E), that is, 111.1 µN (170°) < 126.1 µN (160°) < 128.3 µN (150°) < 135.4 µN (140°) < 146.2 µN (130°) < 153.1 µN (124°). The changing trend also perfectly matches that of deposition efficiency (Figure 2E). Overall, the denser and more crowded coacervate systems are more favorable for the enhancement of adhesion force and the high‐efficient deposition on the widespread plant foliage.

Indeed, the significantly enhanced deposition originates from the dense nanosized network‐like microstructure of the coacervate. The resultant pinning of the coacervate on the substrates is approved by the SEM and cryo‐SEM images of the NaDC/DOAB coacervate droplets after bottom‐up impact (Figure 3F,G). The images show that the interlaced fibrous micro/nanostructures of hydrophobically modified filter paper are fully or partially covered by the thin layer of the coacervate. On the smaller scale, the freeze fractured and sublimed images shows that the spherical coacervate microdroplets firmly adhere to the nanofibers of the filter paper surface and the surfaces between the nanofibers, inducing the wetting transition from Cassie state to Wenzel state. When the droplet is impacted on the back, the droplet's gravity direction is completely opposite to the impact direction, which makes the deposition exceedingly difficult. In contrast, the coacervate with a highly compacted network‐like internal structure can entangle with the micro/nanostructures of superhydrophobic surface, ensuring a strong adhesion with such surfaces and dramatically inhibiting the downward rebounding behavior even after bottom‐up spraying. Moreover, the denser network‐like structure of coacervate is more beneficial for the entanglement with the micro/nanostructure on the hydrophobic/superhydrophobic substrates because the denser structure may more closely match the micro/nanostructure of the surface in size. As a result, the high deposition efficiency of sprayed droplets on the inverted hydrophobic/superhydrophobic surfaces is achieved by overcoming the gravity of droplets.

**Figure 4:**
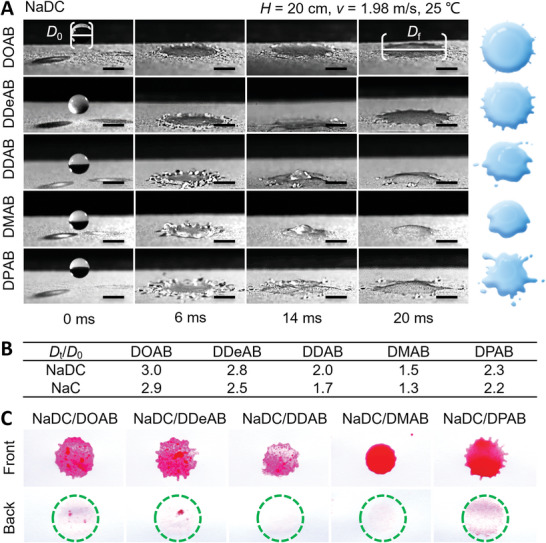
High‐speed impact of coacervate droplets on a superhydrophobic surface. A) Side views of the impact dynamics of 30 mm NaDC/30 mm DXAB coacervate droplets on superhydrophobic surfaces (*θ* = 150°), and schematic illustrations of the final state of the droplets. The impact velocity is 1.98 m s^−1^. Scale bar, 2 mm. B) The spreading factor *D*_t_/*D*_0_ on the superhydrophobic surface for 30 mm NaDC/30 mm DXAB and 30 mm NaC/30 mm DXAB coacervate droplets, *D*_0_, initial droplet diameter, *D*_t_, the spreading area diameter at 20 ms. C) Front and back views of the impact results of 30 mm NaDC/30 mm DXAB coacervate dispersions on the same superhydrophobic paper. In all images, DXAB = DOAB, DDeAB, DDAB, DMAB, and DPAB.

However, the impacting dynamics of water droplets on the underside cannot be clearly recorded by a high‐speed camera. In order to further know the deposition and spread of the coacervate droplets in detail, we observe the dynamic impacting process of the droplets (similar impact velocity of 1.98 m s^−1^) on the upper superhydrophobic surface with a contact angle of 150° (**Figure** **4**A; Figure S5 and Movie S2, Supporting Information). Strikingly, different from single NaDC, NaC, and DXAB which show incomplete rebound off the surface (Figure S6, Supporting Information), there is no splash and rebound and restriction occurring for the coacervates with the shortest surfactant DOAB. Moreover, the edge of droplets is stable and there are no obvious finger droplets and droplet fragmentation in the whole spreading process. With increasing the surfactant alkyl chain length, the droplets splash and split more strongly, and start to rebound after reaching the maximum spreading diameter in 6.0 ms. For the coacervate with the longest surfactant DPAB, an exception appears again, that is, its droplet shows partial spread or splash on the surface. The impact behaviors are also reflected in the average diameter ratio (*D*_t_/*D*_0_) of the final spreading liquid to the initial droplet (Figure 4B). It demonstrates that network‐like microstructure enables the coacervate droplets to entangle with the micro/nanostructures of a superhydrophobic surface, and the more crowded one has the stronger ability to efficiently inhibit rebound and spread on a superhydrophobic surface after high‐speed impact.

As a pesticide carrier, the penetrability of the modified droplets on the surfaces is another key property of the application. Taking the superhydrophobic filter paper with a 150° water contact angle to mimic leaf surfaces, the front and back outcomes of the impacted droplets apparently confirm that all the rhodamine 6G‐encapsulated droplets deposit and spread on the superhydrophobic paper, but only the NaDC/DOAB, NaDC/DDeAB, and NaDC/DPAB droplets penetrate the paper no matter which is faceup or inverted (Figure 4C and Figure S7, Supporting Information). It confirms that the coacervate with a higher crowding degree not only provides the stronger adhesion of coacervate at the liquid/solid phase interface to enhance surface energy but also subsequently improves its penetrability.

Overall, the impact and penetration behaviors on the superhydrophobic surface are broadly consistent with the deposition efficiency on the inverted superhydrophobic surface. Adjusting the compactness of the nanosized network‐like microstructure of coacervates can significantly improve the deposition and penetration on both adaxial and abaxial sides of plant leaves, after either bottom–up or top–down spraying.

**Figure 5:**
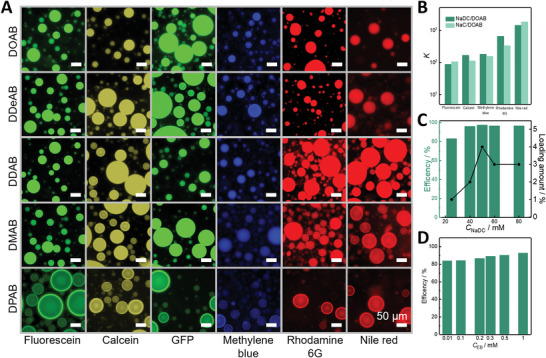
Sequestration of hydrophilic/hydrophobic dyes, biomacromolecule and pesticide in NaDC/DXAB coacervates. A) Confocal fluorescence images of anionic dyes (fluorescein, calcein), cationic dyes (methylene blue and rhodamine 6G), hydrophobic dye (Nile red), and biomacromolecules (GFP) encapsulated by 30 mm NaDC/30 mm DXAB. The dye concentration is 0.03 mm. DXAB = DOAB, DDeAB, DDAB, DMAB, and DPAB. Scale bar, 50 µm. B) Partition coefficients of the dye sequestration within coacervates in 30 mm NaDC/30 mm DOAB and 30 mm NaC/30 mm DOAB. UV–vis absorption spectra of both the supernatant and coacervate phase were measured after phase separation. C) Encapsulation efficiency of 10 µm emamectin benzoate by NaDC/DOAB at a molar ratio of 1:1 and different NaDC concentrations. D) Encapsulation efficiency of emamectin benzoate (≈0.01–1.0 mm) by 30 mm NaDC/30 mm DOAB coacervate dispersions.

**Figure 6:**
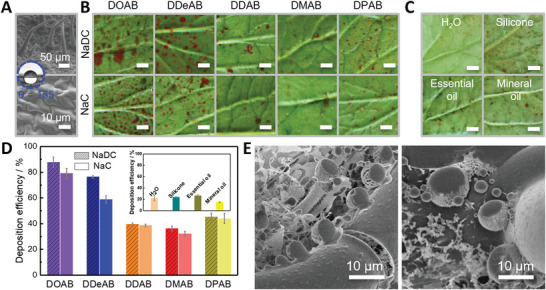
Evaluation of the coacervate properties for practical application. A) SEM image of the superhydrophobic underside of tomato leaves with a water contact angle of 155^o^. B,C) Optical images of the abaxial side of tomato leaves on which coacervate droplets (B) and pure water (C), three types of commercial agricultural adjuvants (1.3 mg L^−1^ silicone, 1.0 mg L^−1^ essential oil, and 1.0 mg L^−1^ mineral oil) with red dye deposited after bottom‐up spraying. The coacervates are formed by 30 mm NaDC/30 mm DXAB and 30 mm NaC/30 mm DXAB. DXAB = DOAB, DDeAB, DDAB, DMAB, and DPAB. Scale bar, 0.5 cm. D) The deposition efficiency of emamectin benzoate on the abaxial side of tomato leaves which are analyzed by high‐performance liquid chromatography (HPLC). E) Cryo‐SEM image of coacervates droplets (30 mm NaDC/30 mm DOAB) after impacting on the abaxial side of tomato leaves.

On the basis of the above results, we further evaluate the practical application of the coacervates (**Figures** **5** and **6**). First, the encapsulation ability of the coacervates to pesticides is evaluated. The various fluorescent dyes were selected to represent the pesticides of different water solubility and molecular weight, including cationic dyes (methylene blue and rhodamine 6G), anionic dyes (fluorescein and calcein), hydrophobic dye (Nile red) dyes and biomacromolecule (GFP). As we expected, both hydrophobic and hydrophilic molecules are preferentially uptake and enriched in the coacervates, because of the intrinsic network‐like microstructure rich in both hydrophilic interfaces and hydrophobic microdomains (Figure 5A). For the best deposition coacervate systems, that is, NaDC/DOAB and NaC/DOAB, the partition coefficients (*K*) of the dyes in the two phases were quantified by UV–vis absorption spectroscopy (Figure 5B), and the results indicate that the hydrophobic dyes are most strongly sequestered into the coacervates (*K* > 1000), whereas the positively and negatively charged dyes are lower but still significant partitioned into the coacervates (*K* is between 100 and 800). Meanwhile, the partition coefficients of GFP were estimated by the ratios of the fluorescence intensities inside and out of the coacervate microdroplets, and the *K* values were all larger than 1000, suggesting that the biomacromolecules were also sequestrated into the coacervates. Combined with the high encapsulation capability of other coacervate systems (Figure 5A), these coacervates are approved to be suitable carriers for various pesticides.

Furthermore, the encapsulation capability of the coacervates to a hydrophobic pesticide is evaluated. Hydrophobic agrochemicals are the most widely used, but their encapsulation efficiency is normally too low to reach their optimum concentration, especially in aqueous media. Here, emamectin benzoate (EB) is a typical hydrophobic pesticide and is widely used to control cotton ling, mites, coleoptera, and homopteran pests.^[^ ^15^ ^]^ By adding EB to the coacervate dispersions, the EB‐encapsulated coacervates are achieved without any organic solvent (Figure S8, Supporting Information). The encapsulation efficiency of EB in the NaDC/DOAB coacervate determined by UV–vis intensity indicates that EB preferentially partitions into the coacervate phase (Figure 5C). Even when the EB concentration was as low as 10 µm, the encapsulation efficiency reaches ≈83–96% for NaDC/DOAB coacervate with equal stoichiometry and varying NaDC concentration from 25 to 80 mm. For the 30 mm NaDC/30 mm DOAB coacervate dispersion, the high encapsulation efficiency is maintained even if the EB concentration is increased up to 1.0 mm, which is much higher than the pesticide concentration normally used (Figure 5D). In principle, the EB molecules may mainly distribute in the hydrophobic domains composed of the bile salt backbone and the hydrophobic chains of surfactants, thereby the present coacervate is also able to encapsulate hydrophobic pesticides and a good carrier for pesticides.

In order to approve the practical effects of the coacervate in depositing on the abaxial side of leaves after bottom‐up spraying, tomato is taken as a representative. Tomato is the most common vegetable in the world and often suffers from pests and diseases on the abaxial side of leaves.^[^ ^3^ ^]^ The abaxial side of tomato leaf is of high superhydrophobicity (*θ* = 155° ± 1°, Figure 6A) because of the presence of flagellate and oblate papillary complex structure, making the deposition more difficult, and its adaxial side is also highly hydrophobic (*θ* = 130° ± 1°, Figure S9, Supporting Information). After bottom–up spraying to tomato leaves, both the imaged deposition results (Figure 6B,C) for the dye‐encapsulated coacervates and the retention efficiency of EB quantitively determined by HPLC (Figure 6D and Figure S10, Supporting Information). It indicates that the coacervates significantly enhance the deposition on the abaxial side of tomato leaves, which is consistent with the results on the artificial superhydrophobic substrate (Figure 2). The NaDC/DOAB coacervate shows the highest efficiency, up to 87.7%, much higher than water and the three typical commercial agricultural adjuvants (≈14.7–26.0% for silicone, essential oil, and mineral oil, Figure 6C,D). When the selected leaf undersides are lowly hydrophobic or hydrophilic, for example, capsicum and corn (*θ* = 97^o^ and 47^o^), the deposition efficiency is apparently much higher than that on the superhydrophobic abaxial‐side of tomato leaves, but their changing tendency is broadly consistent (Figure S11, Support Information). It demonstrates that the NaDC/DXAB and NaC/DXAB coacervates are favorable to enhance the pesticide deposition on abaxial‐side for a broad range of plant leaves. As presented in the freeze fractured and sublimed images under cryo‐SEM, the specific coacervate droplets intactly bind to the flagellate‐like structure of the tomato leaves or the area around its root, while the oblate papillary structure is crowdedly covered by the thin liquid layer of the coacervate (Figure 6E), similar to the observation on the superhydrophobic filter paper after spraying (Figure 3G). That is, the higher deposition of sprayed droplets against rebound and gravity stems from the dense nanosized network‐like structure of coacervate, and the resultant strong pinning between the coacervate and superhydrophobic surface.

**Figure 7:**
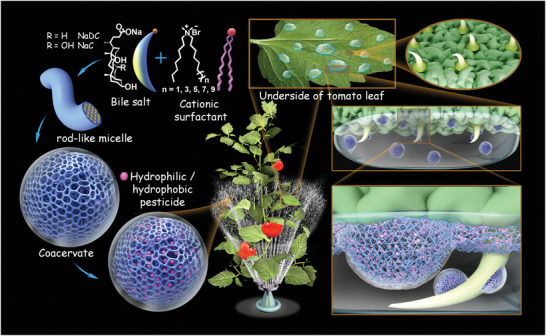
Schematic of the strategy for controlling water‐based pesticide spray on the superhydrophobic abaxial side of tomato leaves. The mixtures of NaDC/NaC and DXAB self‐assemble into rodlike micelle and then coacervate with nanosized network‐like microstructure. The hydrophilic or hydrophobic pesticide is easily and high‐efficiently encapsulated in coacervate. The pesticide‐encapsulated coacervate remarkably improves the deposition of water‐based droplets on the hydrophobic/superhydrophobic abaxial side of leaf after bottom‐up spraying. The deposition efficiency depends on the hydrophobicity degree of the leaf surface and the compactness of the nanosized network‐like microstructure adjusted by the alkyl chain length of the surfactants. This innovative pesticide carrier provides a new and feasible strategy to address the challenges of pesticides with low spray efficiency on the hydrophobic/superhydrophobic abaxial side of plant leaves.

The mixtures of bile salts (NaDC/NaC) and dialkyldimethylammonium bromides with alkyl chain length (DXAB) show a strong ability to form rodlike micelles and coacervates in aqueous solution, resulting from the combination of electrostatic attraction between the oppositely charged headgroups of the two surfactants, the hydrophobic interaction among the hydrophobic parts of NaDC/NaC and multiple alkyl chains of DXAB, and the hydrogen‐bonding interaction between the hydroxyl groups of bile salts (**Figure** **7**). The coacervate droplets contain the nanosized network‐like microstructures and the hydrophilic/hydrophobic domains, so the coacervates facilely encapsulate a broad range of solutes, and exhibit superior affinity to the micro/nanostructures of both the superhydrophobic plant leaves and the filter paper surfaces ranging from hydrophobic to superhydrophobic. The coacervate structural feature promotes the wetting transition from Cassie state to Wenzel state, and endows the coacervate with high‐efficient deposition on such surfaces after high‐speed impact. Moreover, although the viscosity of the condensed phase is enhanced with increasing the alkyl chain length of DXAB, the nonmonotonic changes in the compactness of network‐like structure take a significantly important role in its adhesion force and deposition efficiency of coacervates on the hydrophobic/superhydrophobic surfaces after bottom‐up and top‐down spraying. With the addition of extremely low viscosity without any organic solvents, the coacervates can be used as a high‐efficient pesticide carrier, which is suitable for both the adaxial and abaxial sides of plant leaves of various hydrophobicity. Our method provides a feasible and targeted strategy for the pesticide carriers, especially aimed at the bottom‐up spray for the thick canopy plants with multiple layers of leaves against downward rebound and gravity.

## 3 Conclusion

To sum up, we have first proposed a general approach to finely adjust crowding inside coacervate microdroplets by changing the surfactant chain length, and found the compactness of network‐like structures in coacervates can significantly improve dynamic deposition on inverted hydrophobic/superhydrophobic surfaces. The crowding relies on coacervates prepared by mixing oppositely charged bile salts and quaternary ammonium surfactants in an aqueous solution. The strong entanglement of the extensive nanoscale network‐like structures in the coacervate microdroplets with the micro/nanostructures of hydrophobic/superhydrophobic surfaces overcomes the downward rebound and gravity, thus leading to a very high deposition efficiency of droplets on the undersides, much higher than that of pure water, single component and commercial agricultural adjuvants. Remarkably, the adhesive force and deposition efficiency perfectly correspond to the changing trend of the density of the condensed phase, as well as with increasing the alkyl chain length of surfactants. It demonstrates that the crowding degree is a key factor in enhancing the deposition, and the most crowded one leads to the best deposition. Meanwhile, the various solutes can be highly sequestrated in these coacervates due to the coexistence of the hydrophilic/hydrophobic domains in the coacervates. As a proof of concept, the pesticide‐encapsulated coacervates also show obviously enhanced deposition efficiency on the abaxial side of tomato leaves after bottom‐up high‐speed spraying, which confirms the potential of coacervates to be superior pesticide carriers, and is expected to save pesticides and improve the yield of crops. In addition, the series of modified substrates of different hydrophobicity degrees provide a more reasonable detective approach, which can test the deposition efficiency targeted on individual foliage of different hydrophobicity. The proposed strategy to improve dynamic deposition by adjusting the crowding inside coacervate droplets would be also promising in other fields, such as spray coating and inkjet printing.

## Conflict of Interest

The authors declare no conflict of interest.

## Supporting information

Supporting Information

Supplemental Movie 1

Supplemental Movie 2

## Data Availability

The data that support the findings of this study are available from the corresponding author upon reasonable request.
