# Supplementary material for: Coacervate‐Enhanced Deposition of Sprayed Pesticide on Hydrophobic/Superhydrophobic Abaxial Leaf Surfaces
Source: Adv Sci (Weinh). 2023 Apr 20;10(18):2300270. doi: 10.1002/advs.202300270 (PMC10288258; doi:10.1002/advs.202300270)
Supplement: Supplementary file 1 — Supporting Information [file ADVS-10-2300270-s002.pdf]

Supporting Information

Coacervate-Enhanced Deposition of Sprayed Pesticide on Hydrophobic/Superhydrophobic Abaxial Leaf Surfaces

Liangchen Zhang, Jie Wang, Yaxun Fan, and Yilin Wang**

Table of Contents

Experimental Section 2

Supplementary Figures.. 7

Supplementary Tables.. 18

Movie Captions.. 20

References... 20

Experimental Section

Materials

Sodium deoxycholate ($C_{24}H_{39}O_4Na$, 99%), sodium cholate ($C_{24}H_{39}NaO_5$, 99%), octadecyltrichlorosilane ($C_{18}H_{37}Cl_3Si$, >95%, OTS) and nile red were purchased from Acros. Dihexadecyldimethylammonium bromide ($C_{34}H_{72}NBr$, >97.0%, DPAB), ditetradecyldimethylammonium bromide ($C_{30}H_{64}NBr$, >97.0%, DMAB), didodecyldimethylammonium bromide ($C_{26}H_{56}NBr$, >98.0%, DDAB), didecyldimethylammonium bromide ($C_{22}H_{48}NBr$, >97.0%, DDeAB), dioctyldimethylammonium bromide ($C_{18}H_{40}NBr$, >98.0%, DOAB) and methyltrichlorosilane (CH_3Cl_3Si, >98%, MTS) were purchased from TCI. Rhodamine 6G, fluorescein calcein, and emamectin benzoate ($C_{56}H_{81}NO_{15}$, 99%, EB) were purchased from J&K (Beijing, China). Methylene blue was purchased from Alfa. Green fluorescent protein (GFP) was purchased from Thermo Fisher Scientific (Beijing, China). n-Hexane (AR) was purchased from Conco De (Beijing, China). Filter paper (medium speed qualitative, 11 cm) was purchased from Xinxing (Hangzhou, China), glass slides were purchased from Feizhou (Yangzhou, China) and nonwovens were purchased from Chuanhua (Hangzhou, China). Agricultural silicone additives were purchased from Lvlong (Shandong, China), 24.5% abamectin mineral oil was purchased from Lvheng (Beijing, China) and orange peel essential oil was purchased from Luo Danfeng (Shandong, China). The parts of the spray device were purchased from Dan Meijing (Hangzhou, China) and all the solutions were prepared by Milli-Q (18.2 MΩ·cm).

Phase diagram

The phase boundaries were operationally defined by the critical points obtained from the characterizations of samples at various molar ratios and total concentrations of dialkyldimethylammonium bromide (DXAB = DOAB, DDeAB, DDAB, DMAB, and DPAB) and sodium deoxycholate (NaDC)/sodium cholate (NaC). The test for each mixed sample was repeated at least three times. Micelles were evidenced by cryogenic transmission electron microscopy (JEOL JEM-2010 TEM). Coacervates were confirmed by the phenomenon of phase separation and characterized by optical microscopy (XSP-8C(8CA), Tangxia Xinlei Instruments, Dongguan, China), fluorescence microscopy (Olympus IX83, Olympus, Tokyo, Japan), and cryogenic scanning electron microscopy (S-4300, Hitachi, Ltd., Tokyo, Japan).

Cryogenic Transmission Electron Microscopy (Cryo-TEM). 5 µL solution of micelles (C_{NaDC} = 20 mM, C_{DOAB} = 20 mM) was loaded onto a carbon-coated holey TEM grid and blotted with a filter paper for a few seconds. Then, the grid was quickly plunged into a

reservoir of liquid ethane at −183 °C. The vitrified sample was then stored in liquid nitrogen until it was transferred to a cryogenic sample holder and examined with a JEOL JEM-2010 TEM (120 kV) at about −174 °C. Images were taken by a Gatan multi-scan cooled charge-coupled device (CCD) camera in the minimal electron does mode.

Optical Microscopy. The images of the phase separation were captured by an optical microscope (XSP-8C(8CA)) with a mounted digital camera. Typically, 20 μL of dispersion of coacervate droplets (C_{NaDC} or C_{NaC} = 30 mM, C_{DXAB} = 30 mM) were loaded onto a glass slide and observed using a ×100 lens.

Cryogenic Scanning Electron Microscopy (Cryo-SEM). Coacervates of 30 mM NaDC and 30 mM DOAB were encapsulated in the sample stage and then frozen by plunging into liquid nitrogen slush (−210 °C). Frozen samples were transferred into a cryo-preparation chamber (Quorum 3010T) under vacuum, freeze-fracture, and sublimed at −90 °C for about 15 min. After that, the frozen surface of the samples was coated with tungsten to make it conductive under an argon environment (10 mA for 60 s). Then samples were transferred to a −175 °C cryo-stage in the microscope (Helios Nanolab G3CX), and images were obtained using a 3.0 kV landing energy and 10 μA electric current while keeping a working distance of 4 mm.

Preparation and characterization of hydrophobic/superhydrophobic surfaces

Three types of protocols for coating solutions were used to prepare the filter paper with different contact angles of pure water. (I) For the filter paper with a contact angle of 170° ± 1°/160° ± 1°/150° ± 2°: add 20, 50, and 10 μL of water to 1.0 mL of pure OTS in a 1.7 mL microcentrifuge tube, respectively. The tube was then capped and immediately placed on a vortex mixer for 10 s at 3200 rpm, followed by sonication in an ultrasonic cleaner for 10 s and another round of vortex mixing for 10 s. Immediately after, transfer 500 μL of the resultant emulsion to a 20 mL scintillation vial. After 2 h, add 10 mL of n-hexane to the vial. (II) For the filter paper with a contact angle of 140° ± 2°: the procedure is almost the same as the filter paper with a contact angle of 170° ± 1° except for the last step, *i.e.*, immediately add 10 mL of n-hexane to the vial after transferring 500 μL of the resultant emulsion to a 20 mL scintillation vial. (III) For the filter paper with a contact angle of 130° ± 1°/124° ± 1°: add 10 mL of n-hexane to 100 μL of pure OTS/MTS in a tube and shake to be mixed before use.[1]

As to filter paper surface modification, all solid substrates were immersed in the coating solutions overnight. The treated surfaces were then removed from the solution, washed 3 times with n-hexane, and then air dried. Contact angles were measured by a contact angle device (DSA100, KRUSS) at more than three different locations on the surfaces. The

micro/nanostructure of these surfaces was observed by a field emission scanning electron microscope (SEM) at 10 kV (Hitachi S-4800). The same hydrophobic modification method was used for glass slides and nonwovens.

Deposition efficiency on abaxial side of leaves after bottom-up spraying

A bottom-up spray device was used. The distance between the sprayer and the hydrophobic/superhydrophobic surface was 20 cm, and the volume of sprayed solution was fixed at about 2.0 mL each time. The size of the surface was fixed as an 11 cm diameter circle. After spraying, the surfaces were dried in the air and placed in a vacuum drying oven, which was kept at a constant temperature of 35 °C for 4 h. The net weight of the sprayed solution in the same volume is W_0. Before and after spraying, the weight change of the hydrophobic/superhydrophobic surface is ΔW, so the spray deposition efficiency was calculated by $\Delta W/W_0 \times 100\%$. The deposition efficiency value for each sample was obtained by measuring it at least five times.

Droplet impact on superhydrophobic surfaces

The impacting process of droplets on the surfaces was recorded from the side by a high-speed camera (FASTCAM mini UX100 Photron) with a shooting speed of 2000 FPS/s and a shutter speed of 1/20000 s. The droplet impact velocity was controlled at 1.98 $m\cdot s^{-1}$ by free-falling the droplet from a stainless-steel syringe needle with a settled height (20 cm). The droplet volume was controlled by fixing the inner diameter of the needle with a LongerPump, and the diameter of the droplets was controlled at 2.0 mm.

Adhesion ability test

The hydrophobic/superhydrophobic surface was fixed on the sample stage, and the starting angle of 0° of the turntables was determined in the control panel of the software (LAUDA scientific LSA100). The NaDC/DXAB and NaC/DXAB coacervates droplets were transferred to the surfaces, after setting the target rotation speed to 360° and the acceleration to 10 rounds each time as well as aligning the injection syringe to the position of the selected rotation radius 240 mm. Afterward, the surface spinning was triggered and recorded, and the adhesion force was calculated according to the recorded video file (RFB mode). The adhesion force can be calculated according to Furmidge's equation which is shown below.[2]

$$F = mg\cdot\sin\alpha = \sigma w\ (\cos\theta_R - \cos\theta_A)$$

where, F is the adhesion force, m is the mass of liquid, g is the gravitational acceleration, α is the sliding angle of the droplet, σ and w are the surface tension and the contact circle width of the liquid droplet, θ_R and θ_A represent the receding and advancing contact angles, respectively.

Viscosity test

The bottom phase is termed as the condensed phases for NaDC/DOAB, NaDC/DDeAB, NaDC/DPAB, NaC/DOAB, NaC/DDeAB, NaC/DPAB coacevates, which are required 5 mL at least for the measurement. The rheological properties of the condensed phases of NaDC/DXAB and NaC/DXAB were determined by a ThermoHaake RS300 Rheometer in a 35 mm flat-plate geometry at 25.00 ± 0.05 °C. The zero-shear viscosity is measured by controlling the strain to determine the value by extrapolating the viscosity-shear stress curve to the zero-shear rate. Frequency spectra were recorded in the linear viscoelastic regions, and the shear rate ranged from 0.01 to 1000 s^{-1} in the steady-shear measurements. The measurement was carried out at 1 Hz frequency and 0.1 Pa amplitude of for 30 min.

Anton Paar (MCR302) rotary rheometer was used to measure the shear viscosity of 30 mM NaDC/DXAB and 30 mM NaC/DXAB coacervate dispersions by the barrel measurement method. The samples before phase separation were filled in the circular barrel cup to reach the scale mark, and the measuring rotor was lowered until it was completely submerged in the sample. The sample solution required for the barrel mold is 3.5 mL and the shear rate range is from 0.1 to 1000 s^{-1}.

Morphological characterization of droplets on hydrophobic/superhydrophobic surfaces

The NaDC/DXAB or NaC/DXAB coacervate droplets were sprayed to the abaxial side of surfaces adhered on electron microscope sample stage against gravity at a height of 20 cm. The samples were naturally dried and observed on a scanning electron microscope (SEM, S-4800, Hitachi, Ltd, Tokyo, Japan), or quickly cooled in liquid nitrogen and observed on a Cryo-SEM (S-4300, Hitachi, Ltd, Tokyo, Japan). The prepared samples were sublimed at −90 °C for 30 min for the observation by cryo-SEM, and the rest conditions were the same as the above used for coacervate droplets.

Encapsulation efficiency of dyes and emamectin benzoate (EB)

Different fluorescent dyes (0.03 mM), including cationic dyes (methylene blue and rhodamine 6G), anionic dyes (fluorescein), hydrophobic dye (nile red) and green fluorescent protein (GFP) were added to NaDC/DXAB and NaC/DXAB coacervates.

Ultraviolet and visible spectrophotometry (UV-vis). 10 μM emamectin benzoate (EB) was added to NaDC/DOAB coacervates at the NaDC/DOAB molar ratio of 1:1 and the NaDC concentrations of 25 ~ 80 mM. 10 μM ~ 1.0 mM EB was added to 30 mM NaDC/30 mM DOAB coacervate dispersions. The solution was vortexed vigorously and then kept at 4 °C for 24 h. 1 mL of the supernatant was filtered through a 0.22 μm filter membrane, and the absorption intensity of EB was measured by JASCO V-550 UV-Visible spectrophotometer, by which the encapsulation efficiency was calculated.

Confocal Laser Scanning Microscopy (CLSM). Confocal fluorescence images of 10 μL coacervate droplets were taken by CLSM (Olympus FV1000-IX81), equipped with × 100 objective lens. Two or three fields of view were recorded for each experiment and repeated at least three times. Fluorescence molecules are excited by special filters, λ_{ex} = 559 nm for methylene blue, rhodamine 6G, and nile red, while λ_{ex} = 488 nm for fluorescein, calcein, and GFP. The partition coefficients of GFP in the coacervate phase to the dilute external phase were determined by the ratios of the fluorescence intensity between the two phases based on the CLSM images, on which ten regions were randomly chosen in the coacervate and dilute phase, and the average fluorescence intensity was analyzed by software FV10-ASW4.2.

Pesticide retention on abaxial side of tomato leaves

Assays were performed with the bottom-up spray device mentioned above. The pesticide residue on abaxial side of tomato leaves after the bottom-up deposition of the NaDC/DXAB and NaC/DXAB coacervate dispersions, three commercial agricultural adjuvants (silicone, essential oil, and mineral oil), and pure water was analyzed by high-performance liquid chromatography (HPLC, Agilent ZORBAX, Agilent, Santa Clara, USA). The column was an Agilent Eclipse C18 (2.1 × 50 mm, 3 μm). The mobile phase was 15/85 (*v*/*v*) methanol and 0.1% formic acid solution at a flow rate of 0.3 mL/min. The column temperature was kept at 30 °C. The detection wavelength is 260 nm.

Supplementary Figures

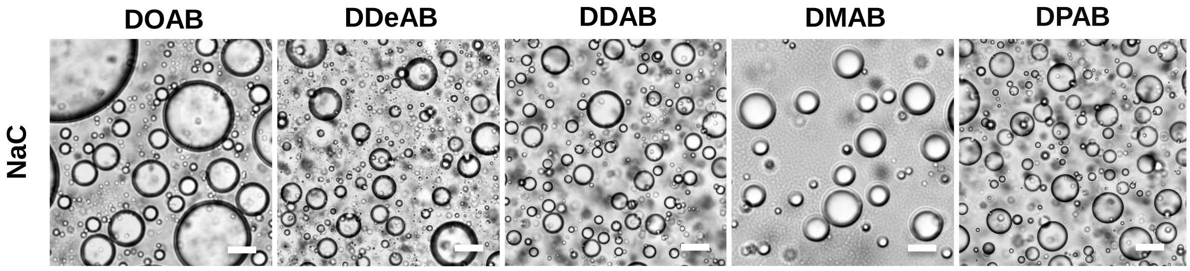

Figure S1. Bright-field microscopy image of coacervate droplets in 30 mM NaC/30 mM DXAB mixtures (DXAB = DOAB, DDeAB, DDAB, DMAB, and DPAB). Scale bar = 20 µm.

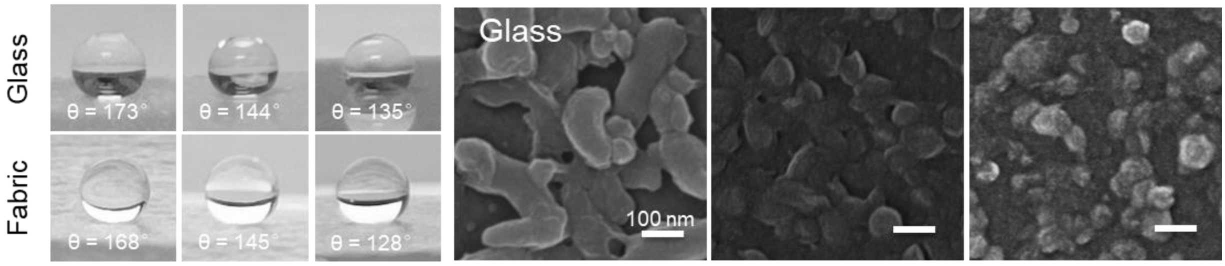

Figure S2. Water contact angle (θ) on modified glass slides and nonwoven fabrics with different hydrophobicity, and SEM images revealing the morphology of the representative surfaces of glass slides (θ = 173° ± 1°, 143° ± 2° and 134° ± 1°). Scale bar for all images = 100 nm.

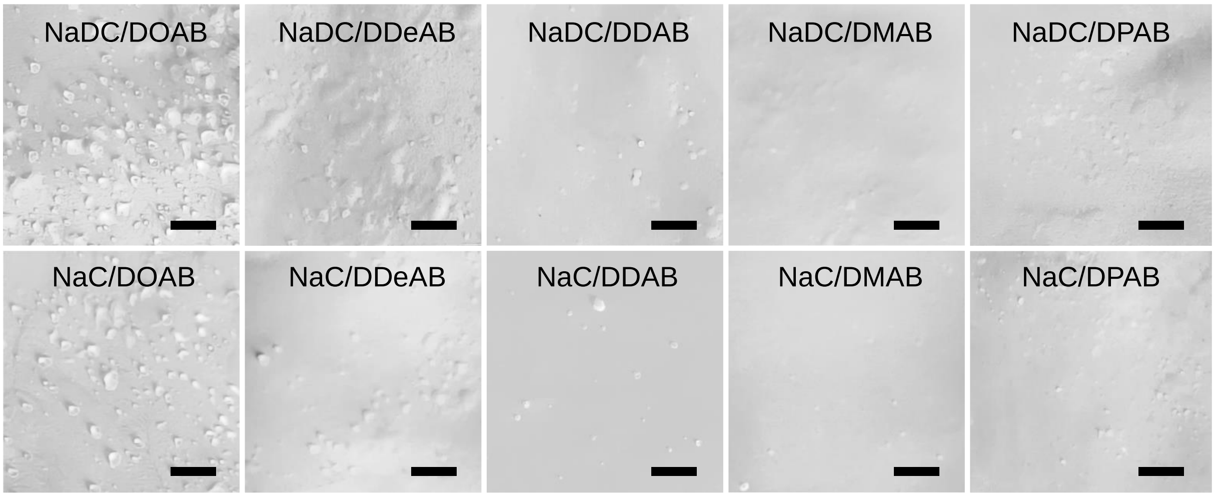

Figure S3. Bright-field optical microscopy images of superhydrophobic filter paper ($\theta = 150°$) on which coacervate droplets are deposited after spraying under bottom-up condition. The coacervates are formed by 30 mM NaDC/30 mM DXAB and 30 mM NaC/30 mM DXAB, DXAB = DOAB, DDeAB, DDAB, DMAB and DPAB. Scale bar for all images = 2 cm.

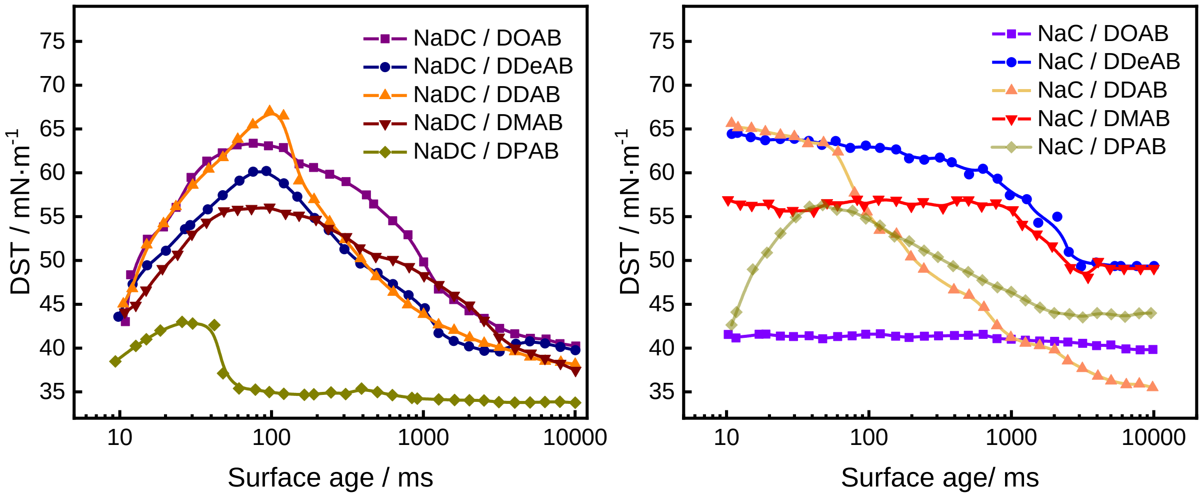

Figure S4. The dynamic surface tension (DST) of 30 mM NaDC/30 mM DXAB and 30 mM NaC/30 mM DXAB coacervate dispersions. DXAB = DOAB, DDeAB, DDAB, DMAB, and DPAB.

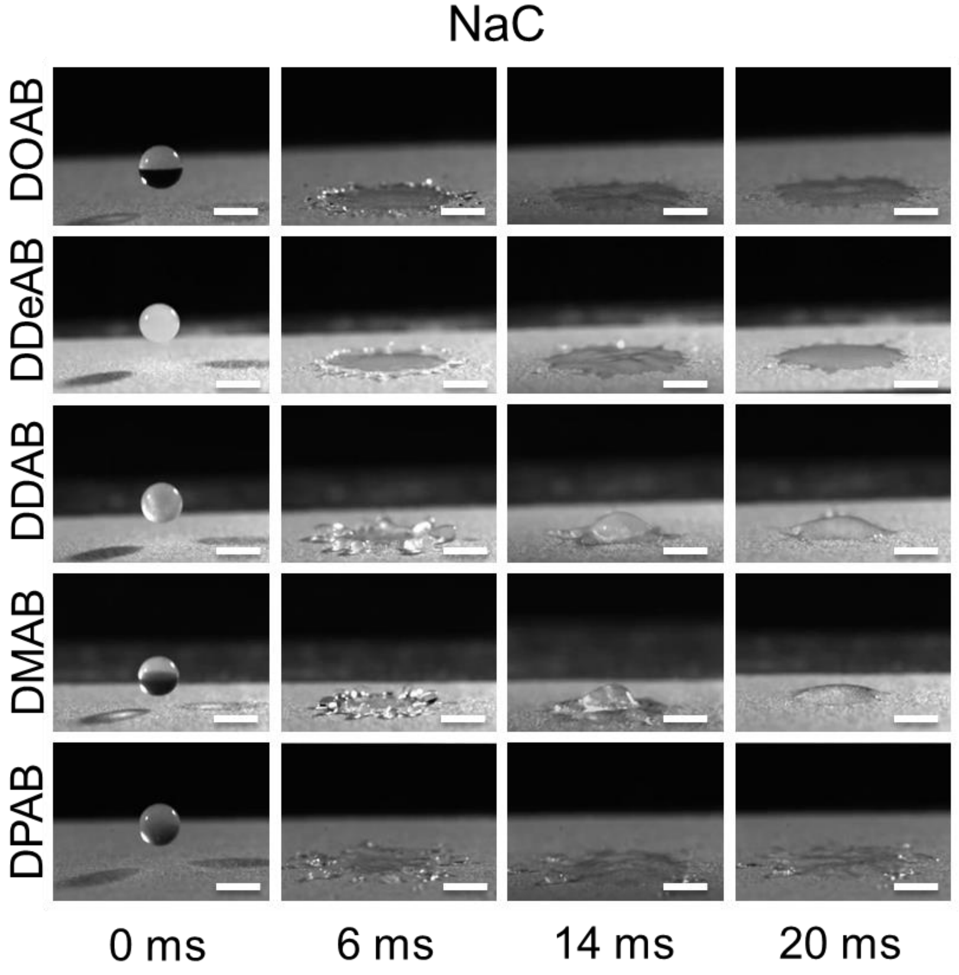

Figure S5. Side views of the impact dynamics of water droplets containing 30 mM NaC/30 mM DXAB coacervate dispersions on superhydrophobic surfaces (θ = 150°). All impact experiments with an impact velocity of 1.98 $m \cdot s^{-1}$. DXAB = DOAB, DDeAB, DDAB, DMAB, and DPAB. Scale bar for all images = 2 mm.

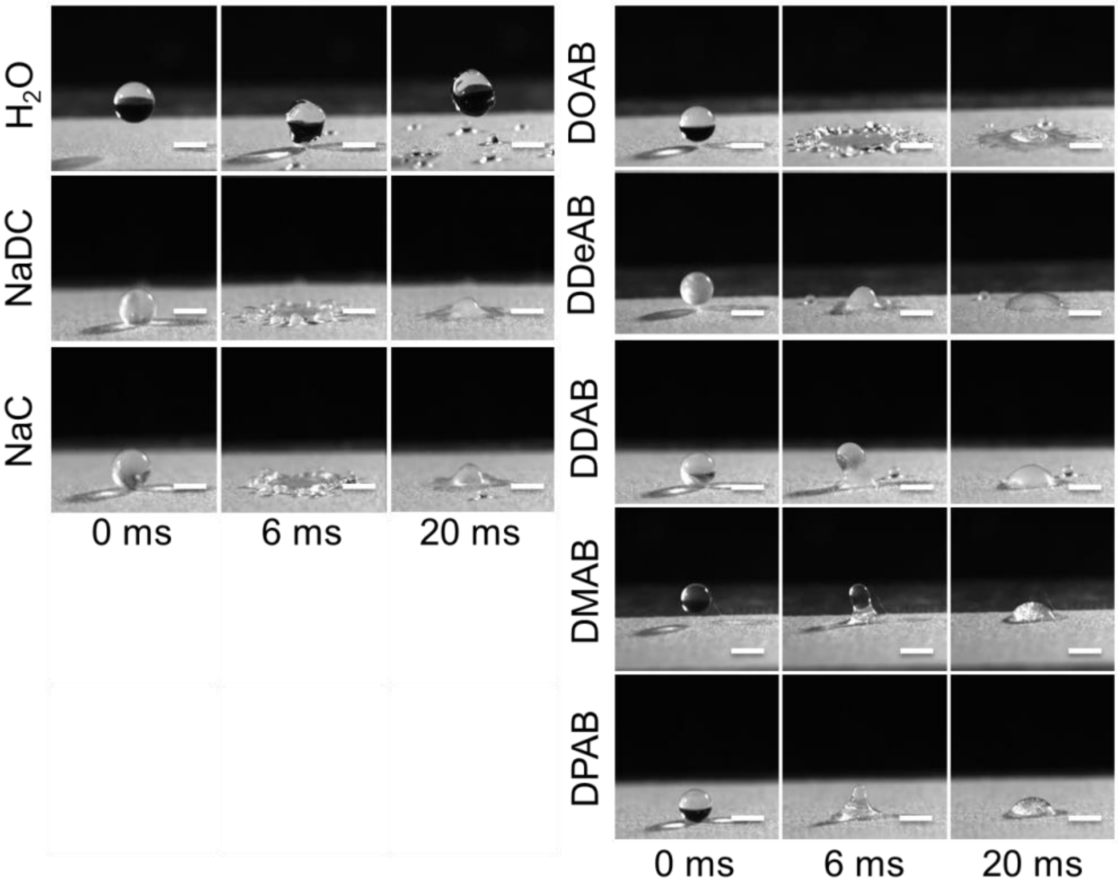

Figure S6. Side views of the impact dynamics of water droplets containing single 60 mM NaDC, NaC and DXAB (DXAB = DOAB, DDeAB, DDAB, DMAB, and DPAB) on superhydrophobic surfaces (θ = 150°), and schematic illustrations of the final state of the droplet. All impact experiments with an impact velocity of 1.98 $m{\cdot}s^{-1}$. Scale bar for all images = 2 mm.

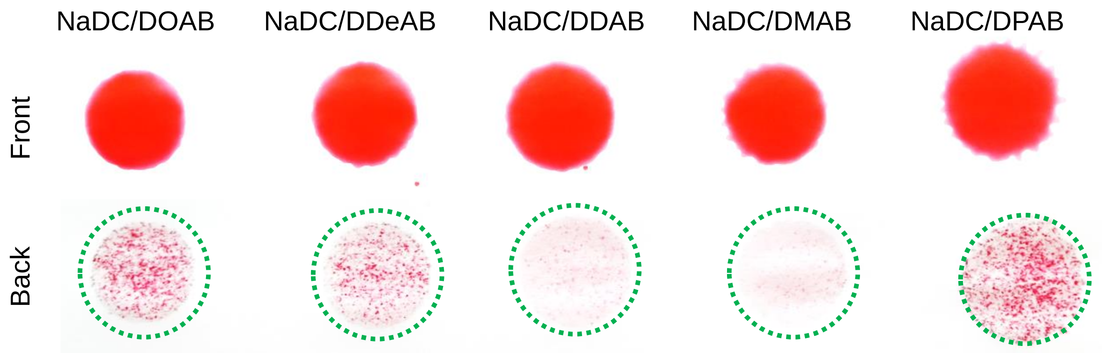

Figure S7. Front and back views of the bottom-up impact results of 30 mM NaDC/30 mM DXAB coacervate dispersions on the same superhydrophobic paper. In all images, DXAB = DOAB, DDeAB, DDAB, DMAB, and DPAB.

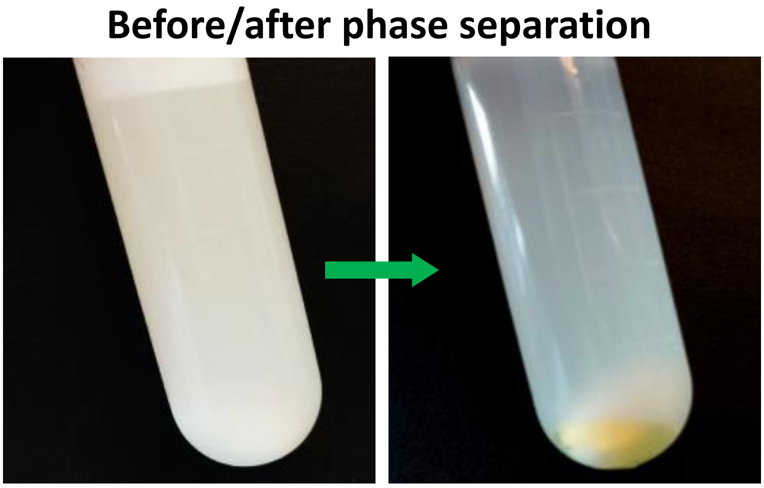

Figure S8. Optical image of 30 mM NaDC/30 mM DOAB encapsulating emamectin benzoate before and after phase separation.

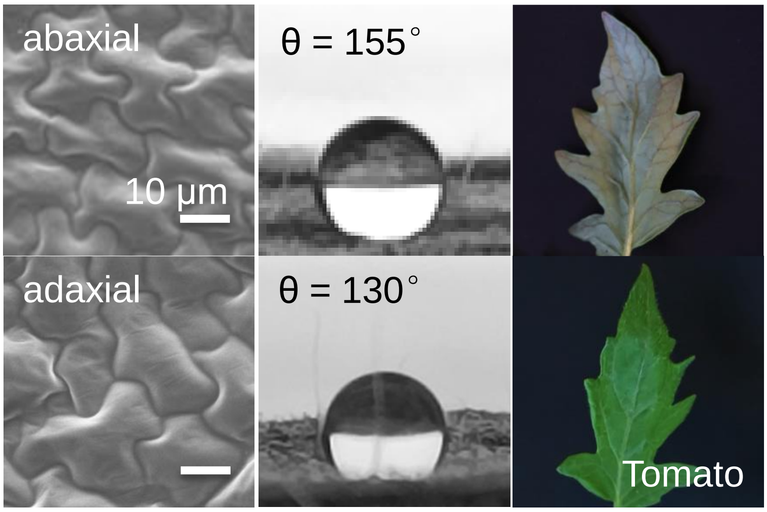

Figure S9. SEM image, water contact angle and macroscopic appearances of the abaxial and adaxial sides of tomato leaves with a water contact angle of 155° and 130°, respectively.

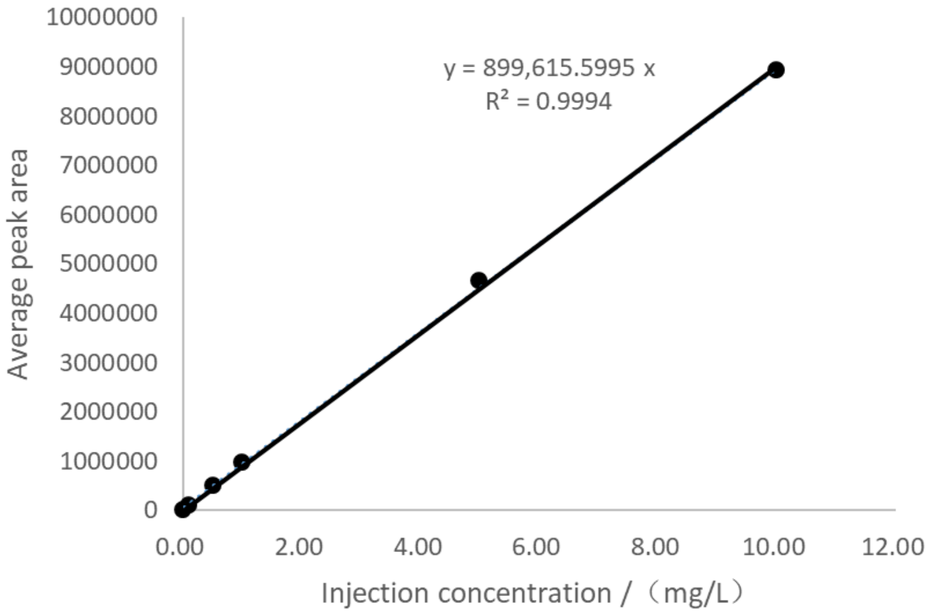

Figure S10. The linear regression equation of standard curve of tomato leaves substrate with emamectin benzoate used to analyze the HPLC data.

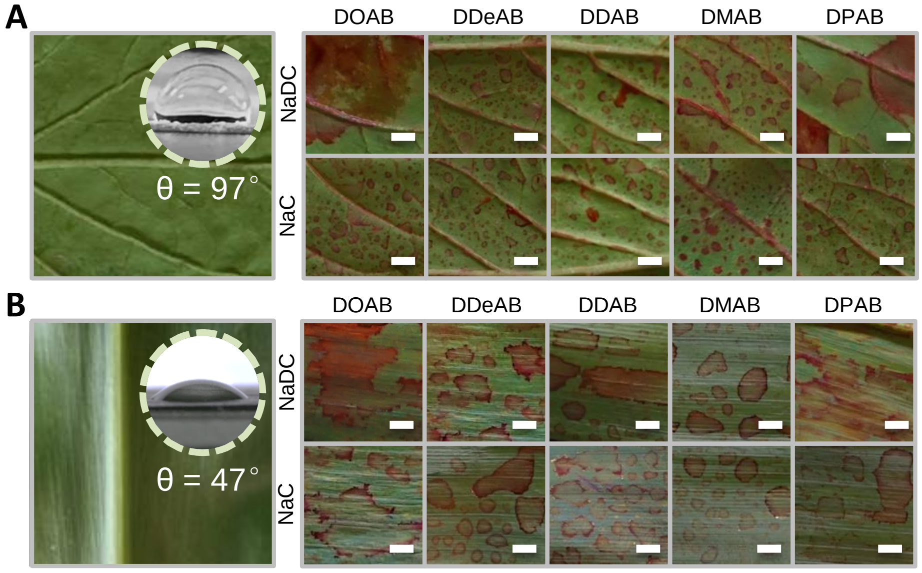

Figure S11. Optical images of the abaxial side of (A) capsicum and (B) corn leaves on coacervate droplets with red dye are deposited after bottom-up spraying. The coacervates are formed by 30 mM NaDC/30 mM DXAB and 30 mM NaC/30 mM DXAB. DXAB = DOAB, DDeAB, DDAB, DMAB and DPAB. Scale bar = 0.5 cm.

Supplementary Tables

Table S1. ζ-Potential and pH values of 30 mM NaDC/30 mM DXAB and 30 mM NaC/30 mM DXAB coacervates. DXAB = DOAB, DDeAB, DDAB, DMAB, and DPAB.

	**NaDC**		**NaC**	
	**pH**	**ζ-Potential/mV**	**pH**	**ζ-Potential/mV**
**DPAB**	8.1	+2.17 ± 0.25	7.8	-6.00 ± 0.21
**DMAB**	7.7	-3.13 ± 0.15	7.6	+5.50 ± 0.20
**DDAB**	8.6	-4.75 ± 0.23	7.6	-6.20 ± 0.19
**DDeAB**	8.6	-1.80 ± 0.20	7.8	-5.50 ± 0.17
**DOAB**	7.8	+0.25 ± 0.06	7.7	-4.30 ± 0.23

Table S2. The weight change of the hydrophobically modified filter paper with different hydrophobicity before and after spraying 2.0 mL coacervate droplets of 30 mM NaDC/30 mM DXAB under bottom-up conditions. The size of superhydrophobic/hydrophobic surface was fixed at 11 cm diameter circle and the value for each sample was obtained by measuring at least three times. DXAB = DOAB, DDeAB, DDAB, DMAB, and DPAB.

**Contact angle/°**		**DOAB /mg**	**DDeAB /mg**	**DPAB /mg**	**DDAB /mg**	**DMAB /mg**
**NaDC**	**170**	7.8 ± 0.3	7.3 ± 0.3	5.9 ± 0.5	4.5 ± 0.3	3.2 ± 0.2
	**160**	13.1 ± 0.3	11.4 ± 0.3	10.7 ± 0.3	9.8 ± 0.2	9.0 ± 0.6
	**150**	18.7 ± 0.5	17.3 ± 0.4	16.4 ± 0.5	14.7 ± 0.4	12.8 ± 0.3
	**140**	22.9 ± 0.3	22.3 ± 0.3	21.8 ± 0.2	18.7 ± 0.3	13.5 ± 0.4
	**130**	27.5 ± 0.3	26.7 ± 0.4	23.6 ± 0.3	20.8 ± 0.2	14.2 ± 0.6
	**124**	34.3 ± 0.6	31.9 ± 0.4	25.7 ± 0.3	21.3 ± 0.3	15.1 ± 0.3
**W_0/mg**		38.6	37.8	37.7	39.9	38

Table S3. The weight change of the hydrophobically modified filter paper with different hydrophobicity before and after spraying 2.0 mL coacervate droplets of 30 mM NaC/30 mM DXAB under bottom-up conditions. The size of superhydrophobic/hydrophobic surface was fixed at 11 cm diameter circle and the value for each sample was obtained by measuring at least three times. DXAB = DOAB, DDeAB, DDAB, DMAB, and DPAB.

Contact angle/°		DOAB /mg	DDeAB /mg	DPAB /mg	DDAB /mg	DMAB /mg
NaC	170	7.4 ± 0.3	6.8 ± 0.3	5.5 ± 0.3	4.1 ± 0.3	2.8 ± 0.2
	160	12.7 ± 0.3	11.0 ± 0.3	10.0 ± 0.5	7.3 ± 0.3	4.1 ± 0.9
	150	18.0 ± 0.6	16.6 ± 0.5	14.6 ± 0.3	12.2 ± 0.3	5.5 ± 0.3
	140	22.4 ± 0.3	21.9 ± 0.3	21.5 ± 0.2	15.4 ± 0.3	7.3 ± 0.4
	130	27.1 ± 0.3	26.0 ± 0.3	25.3 ± 0.3	17.0 ± 0.4	8.5 ± 0.3
	124	33.5 ± 0.5	31.4 ± 0.3	26.5 ± 0.3	19.1 ± 0.4	13.7 ± 0.2
W_0/mg		40	40	39.7	40	37.6

Table S4. The weight change of the hydrophobically modified filter paper with different hydrophobicity before and after spraying 2.0 mL controls (60 mM NaDC, 60 mM NaC, 60 mM DXAB and three types of commercial agricultural adjuvants, including silicone, essential oil and mineral oil) under bottom-up conditions. The size of superhydrophobic/hydrophobic surface was fixed at 11 cm diameter circle and the value for each sample was obtained by measuring at least three times. DXAB = DOAB, DDeAB, DDAB, DMAB, and DPAB.

Contact angle	W_1/mg	W_2/mg	W_0/mg	Contact angle	W_1/mg	W_2/mg	W_0/mg
	170°	124°			170°	124°	
DOAB	1.8 ± 0.1	11.5 ± 0.1	48.5	NaDC	1.9 ± 0.2	9.0 ± 0.1	48.5
DDeAB	2.1 ± 0.1	12.2 ± 0.1	49.0	NaC	1.7 ± 0.1	8.8 ± 0.1	51.0
DPAB	0.8 ± 0.1	4.4 ± 0.3	49.6	Silicone	3.0 ± 0.2	11.8 ± 0.3	55.6
DDAB	2.5 ± 0.1	8.7 ± 0.1	48.5	Essential oil	6.0 ± 0.3	32.4 ± 0.3	92.5
DMAB	2.2 ± 0.1	9.4 ± 0.1	47.4	Mineral oil	5.0 ± 0.2	21.3 ± 0.3	73.5

Movie Captions

Movie S1-S2

Movie S1. Adhesion force of coacervate dispersion droplet on the hydrophobic surfaces. 30 mM NaDC/30 mM DOAB and 30 mM NaC/30 mM DMAB on the superhydrophobic filter paper (θ = 170°). NaDC/DOAB droplet exhibits superior pinning ability on superhydrophobic surfaces. (MP4)

Movie S2. Surfactant coacervates dispersions spray on the hydrophobic surface. 30 mM NaDC (or NaC)/30 mM DXAB droplet impact on the hydrophobic surface (θ = 150°). DXAB = DOAB, DDeAB, DDAB, DMAB, and DPAB. No splash, bounce, or retraction occurred for NaDC/DOAB and NaC/DOAB. (MP4)

References

[1] L. Zhang, A. G. Zhou, B. R. Sun, K. S. Chen, H. Z. Yu, *Nat. Commun.* **2021**, *12*, 982.

[2] Y. Zuo, L. Zheng, C. Zhao, H. Liu, *Small* **2020**, *16*, 1903849.
